# Supplementary material for: Bat Rhinacoviruses Related to Swine Acute Diarrhoea Syndrome Coronavirus Evolve under Strong Host and Geographic Constraints in China and Vietnam
Source: Viruses. 2024 Jul 11;16(7):1114. doi: 10.3390/v16071114 (PMC11281452; doi:10.3390/v16071114)

Supplementary figures

Figure S1. SuperTRI bootstrap 50% majority-rule consensus tree reconstructed from the SWB analysis based on a sliding window of 400 nucleotides moving in steps of 50 nucleotides. The MRP file used for the bootstrap analysis (weighted parsimony, 1000 replicates) includes 1,446,681 characters.

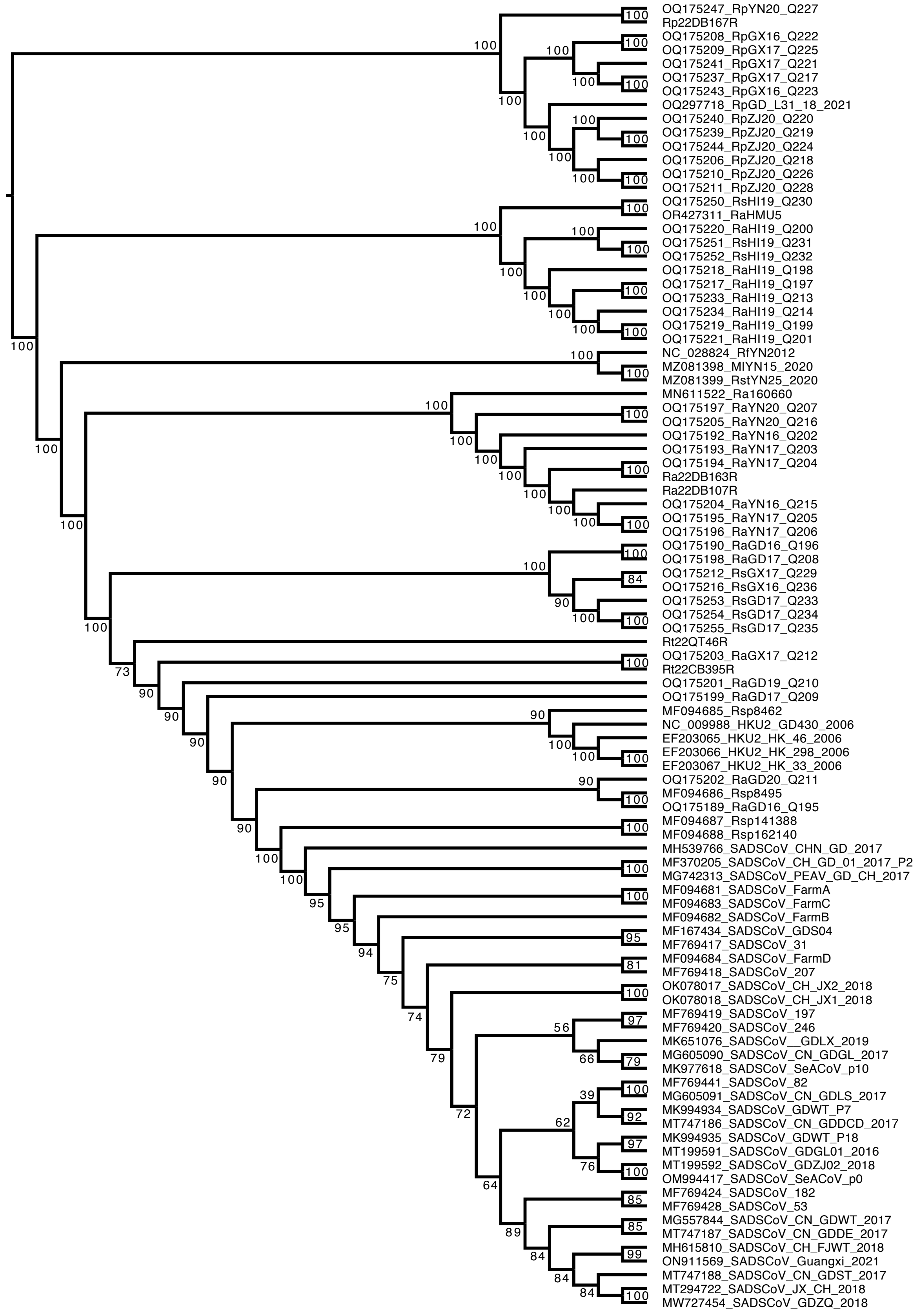

Figure S2. SuperTRI bootstrap 50% majority-rule consensus tree reconstructed from the SWB analysis based on a sliding window of 500 nucleotides moving in steps of 50 nucleotides. The MRP file used for the bootstrap analysis (weighted parsimony, 1000 replicates) includes 1,373,578 characters.

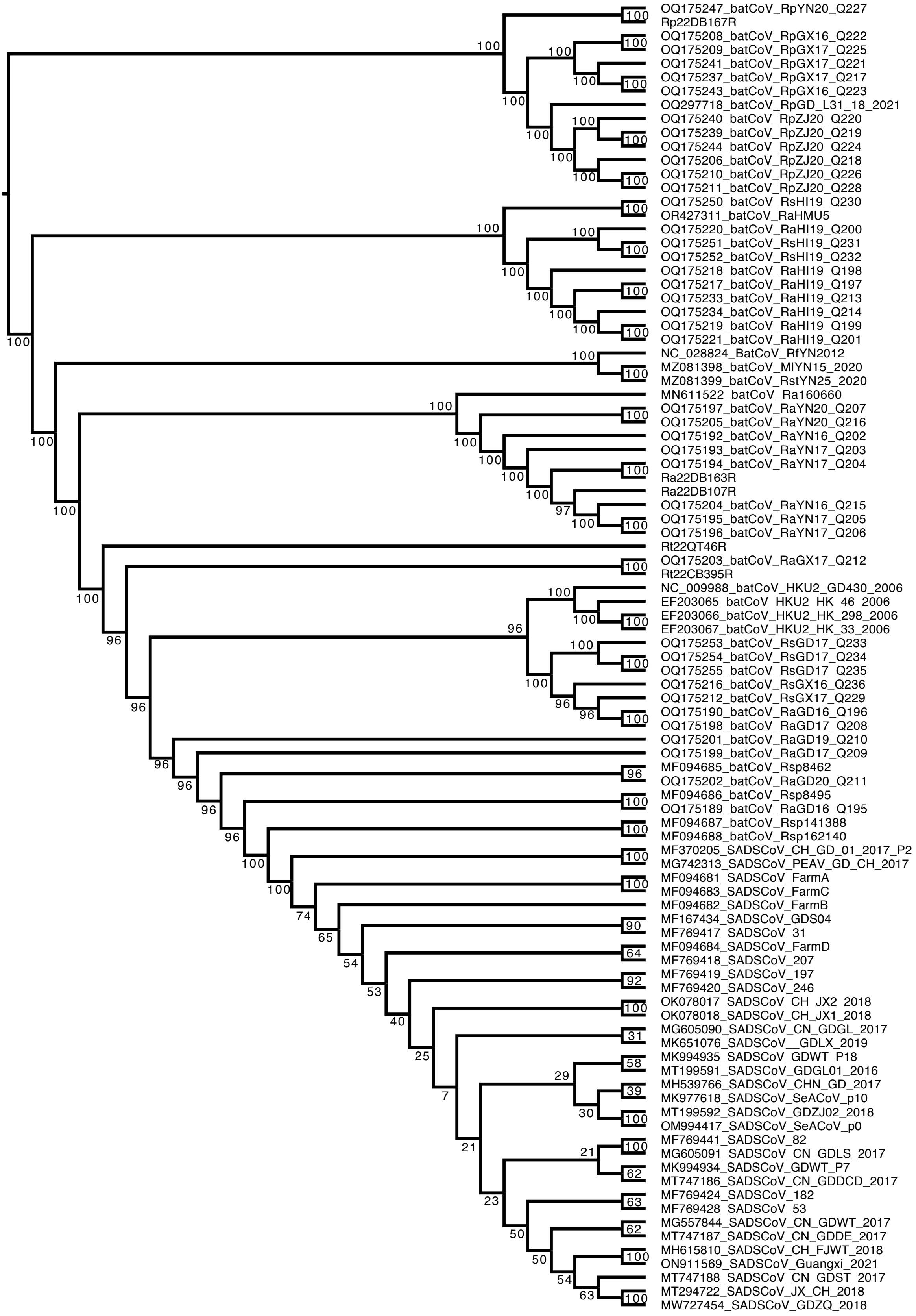

Figure S3. SuperTRI bootstrap 50% majority-rule consensus tree reconstructed from the SWB analysis based on a sliding window of 600 nucleotides moving in steps of 50 nucleotides. The MRP file used for the bootstrap analysis (weighted parsimony, 1000 replicates) includes 1,323,336 characters.

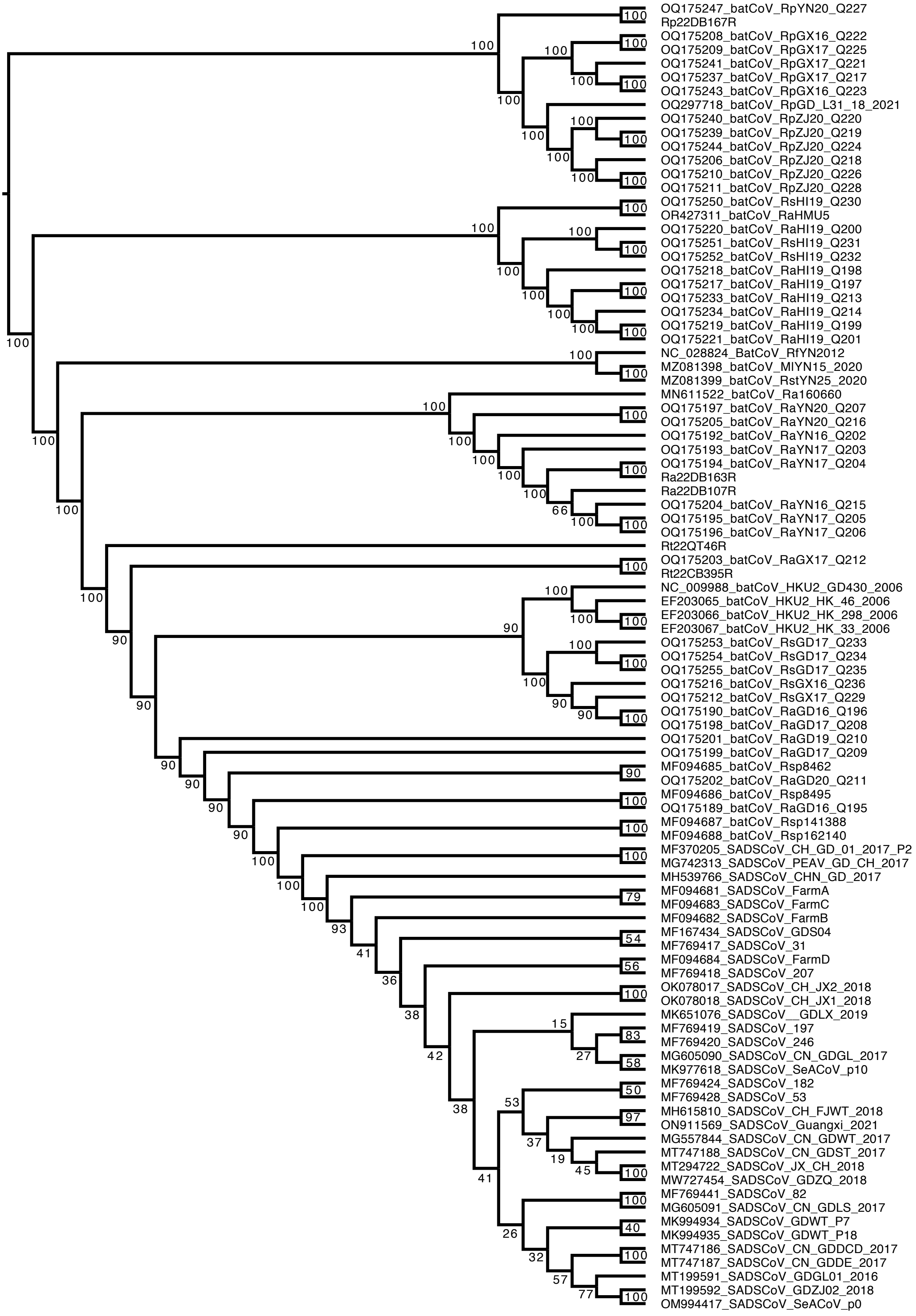

Figure S4. SuperTRI bootstrap 50% majority-rule consensus tree reconstructed from the SWB analysis based on a sliding window of 1000 nucleotides moving in steps of 50 nucleotides. The MRP file used for the bootstrap analysis (weighted parsimony, 1000 replicates) includes 1,186,335 characters.

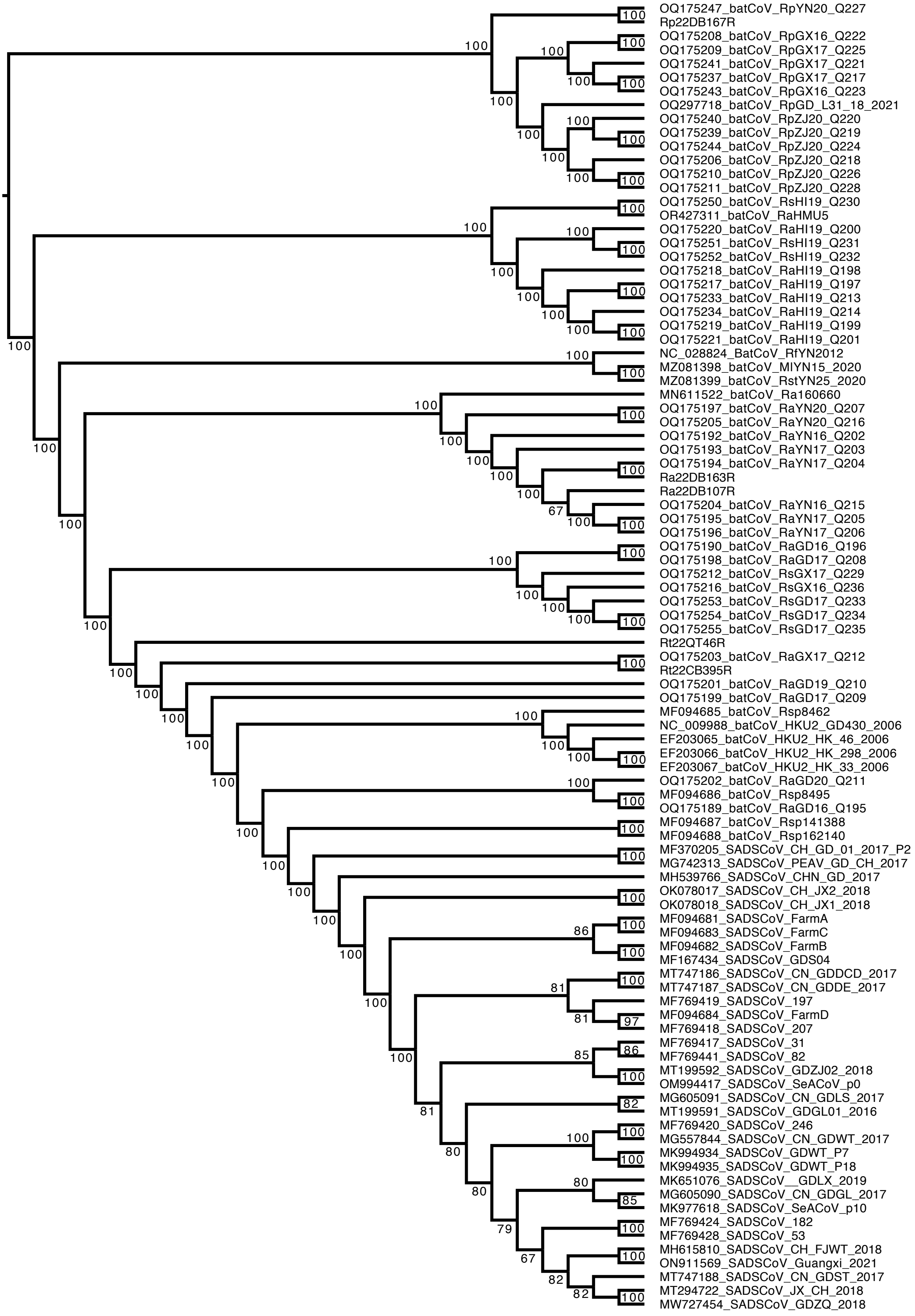

Figure S5. SuperTRI bootstrap 50% majority-rule consensus tree reconstructed from the SWB analysis based on a sliding window of 2000 nucleotides moving in steps of 50 nucleotides. The MRP file used for the bootstrap analysis (weighted parsimony, 1000 replicates) includes 1,020,398 characters.

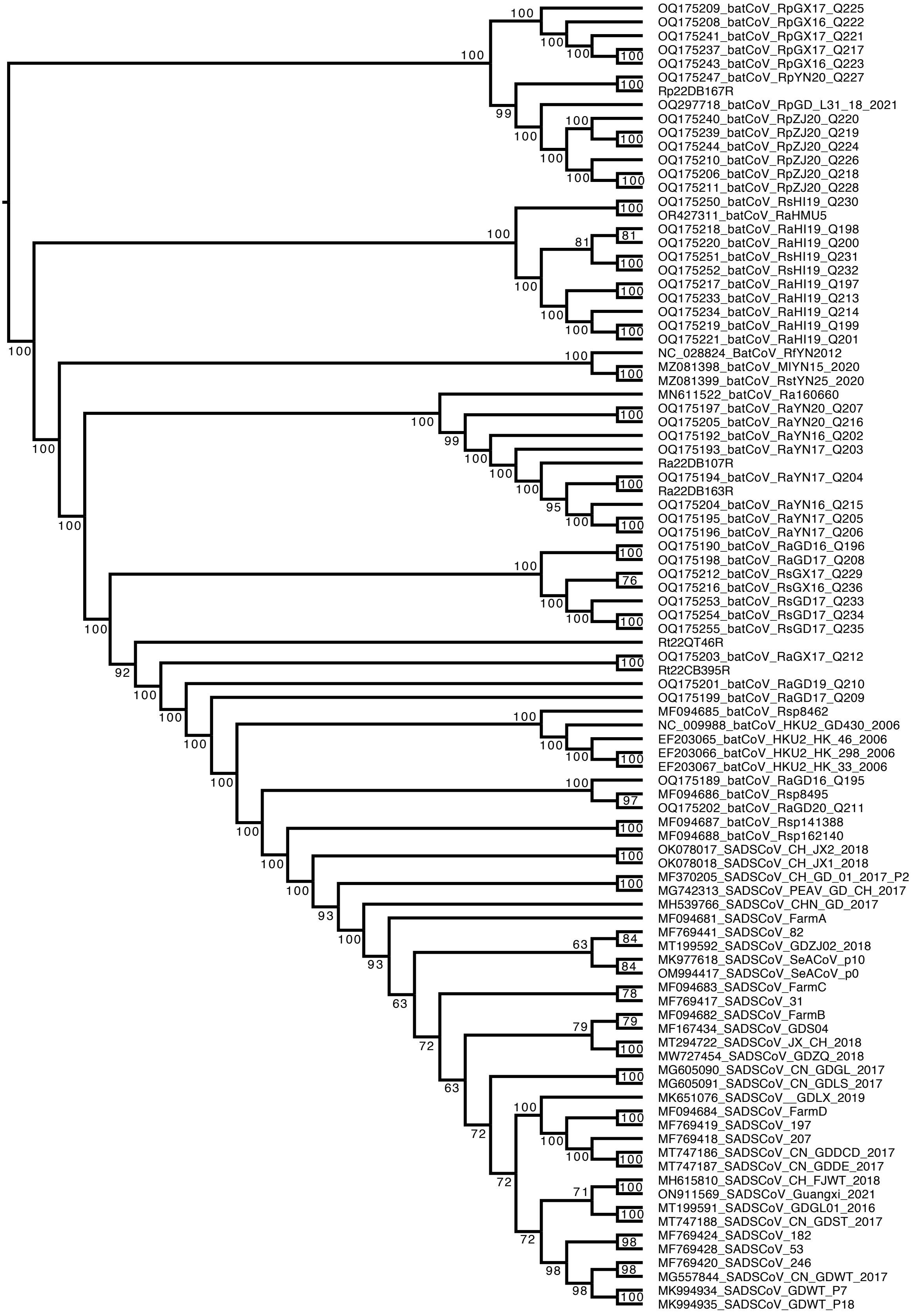

Figure S6. Genomic regions of the three SADS-CoV strains supporting a close relationship with bat rhinacoviruses. The positions of protein-coding genes in the *Rhinacovirus* genome are shown in figure **A**. The five genomic regions found in the three SADS-CoV strains (CH/FJWT/2018, Guangxi/2021, and CHN-GD/2017; GenBank accessions: MH615810, ON911569, and MH539766, respectively) supporting closer relationships with one or several bat rhinacoviruses rather with other SADS-CoVs are shown in figure **B**: in green for CH/FJWT/2018 + Guangxi/2021 (region A: positions 20,101-20,550), dark blue for CH/FJWT/2018 (region B: positions 20,501-22,550; region B': positions 24,001-24,500), and red for CHN-GD/2017 (region C: positions 201-1,600; region D: positions 21,401-21,800).

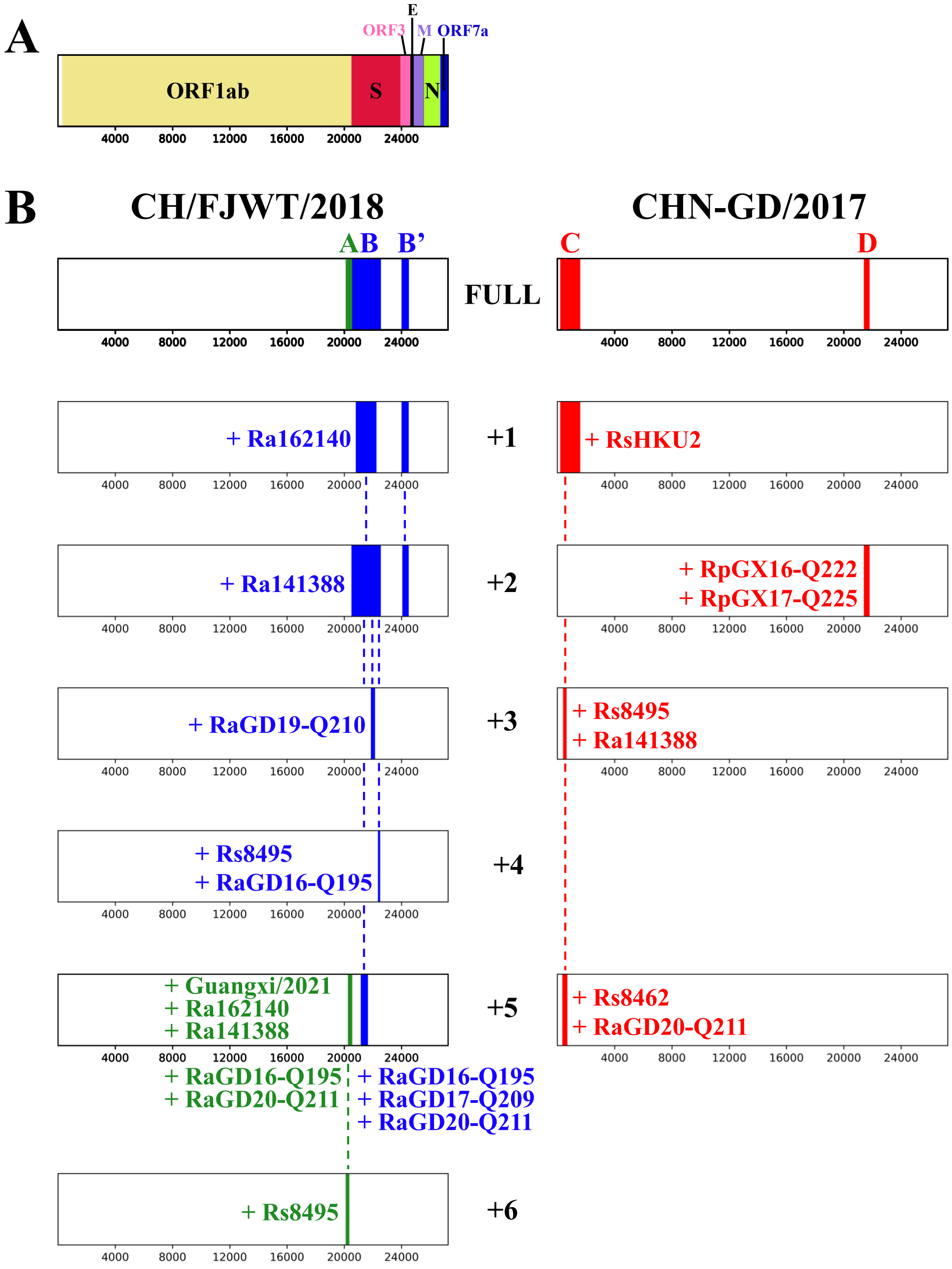

Figure S7. Similarity plot analysis of RaYN20-Q207 *Rhinacovirus* genome. The similarity plot analysis was performed with the SimPlot++ program (Samson et al., 2023) using RaGD17-Q208 (GenBank accession: OQ175198) as reference. It was compared to four *Rhinacovirus* genomes, incuding RaYN20-Q207 (GenBank accession: OQ175197), which was detected as potentially problematic following the study of SWB (Sliding Window Bootstrap) bipartitions (see text for more details). The genomic regions found identical between RaYN20-Q207 and RaGD17-Q208 are highlighted by red bars and their positions in our genome alignment are indicated above.

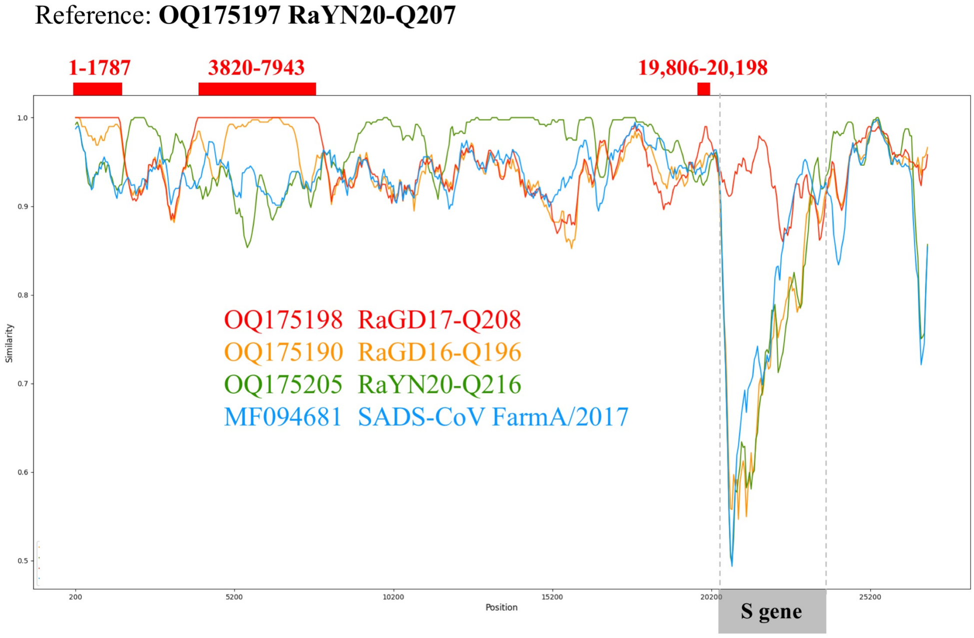

Figure S8. List of autapomorphies found in SADS-CoV genomes based on a strict consensus of 672 most-parsimonious trees.

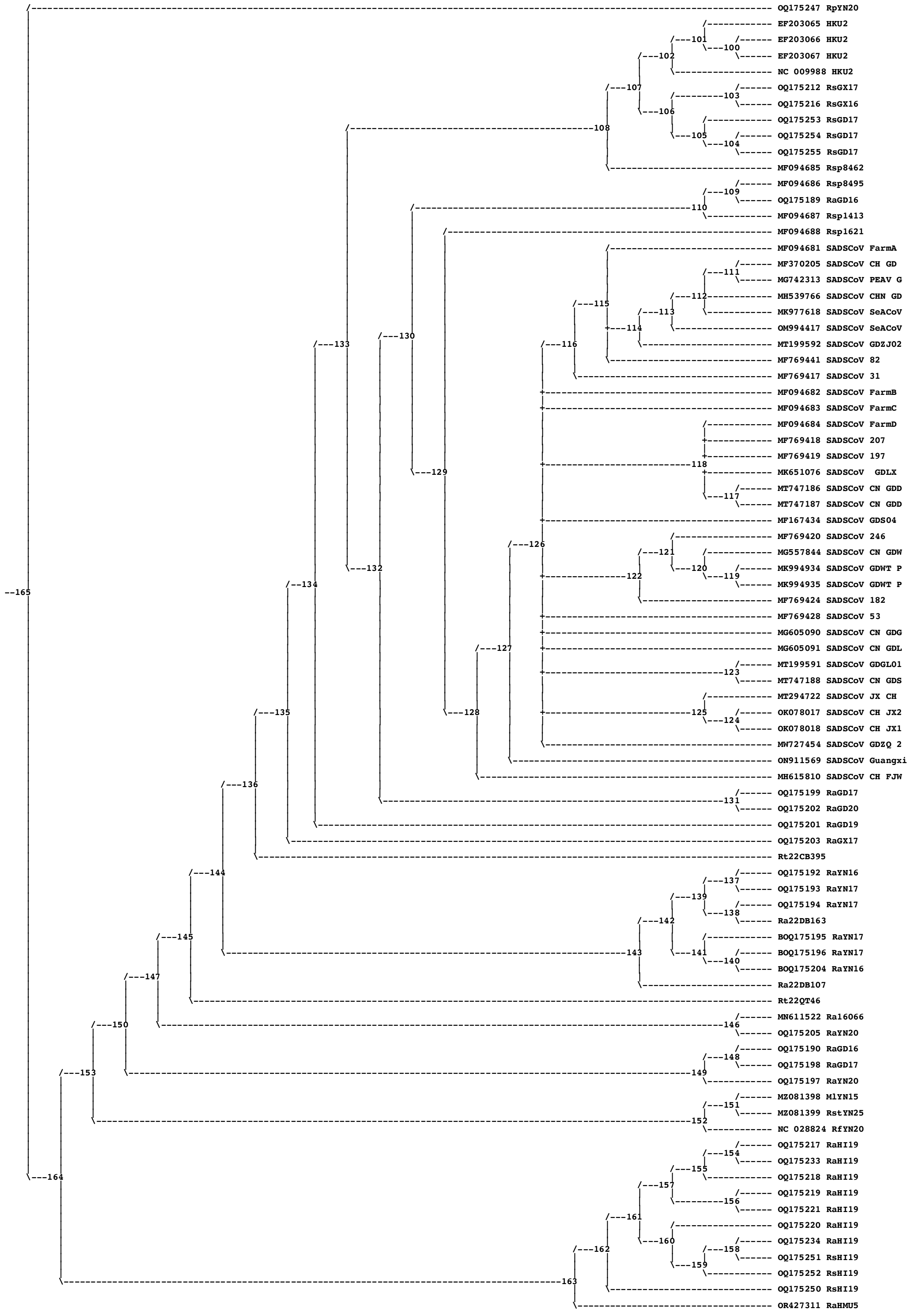

Apomorphy lists:

Branch	Character	Steps	CI	Change
node_111 --> MF370205 SADSCoV CH GD 01 2017 P2	1017	1	0.167	T ==> C

	1607	1	0.500	C ==> T
	3756	1	0.667	T ==> C
	6653	1	1.000	A ==> G
	9323	1	1.000	T ==> C
	10290	1	1.000	T ==> C
	11634	1	0.250	C ==> T
	15102	1	0.143	T ==> C
	22601	1	1.000	C --> T
	22896	1	0.182	C --> G
	22902	1	0.333	A ==> T
	22912	1	0.250	C --> G
	23045	1	1.000	A --> C
	24672	1	1.000	A ==> G
	26235	1	1.000	G ==> A
node_111 --> MG742313 SADSCoV PEAV GD CH 2017	24	1	0.125	T ==> C
	381	1	0.333	T ==> C
	803	1	1.000	A ==> T
	1113	1	0.429	G ==> A
	2108	1	1.000	C ==> G
	2718	1	0.500	T ==> C
	3541	1	1.000	C ==> G
	3950	1	1.000	A ==> T
	4467	1	1.000	T ==> A
	4775	1	1.000	A ==> C
	5244	1	0.667	A ==> T
	7370	1	1.000	T ==> A
	7926	1	0.667	G ==> C
	8597	1	1.000	C ==> G
	11017	1	1.000	C ==> G
	11418	1	0.188	C ==> G
	12435	1	0.500	G ==> A
	13582	1	1.000	T ==> A
	14478	1	0.600	A ==> C
	15769	1	1.000	G ==> A
	17454	1	1.000	A ==> C
	17703	1	0.667	C ==> G
	19195	1	1.000	G ==> C
	19507	1	1.000	T ==> A
	19988	1	1.000	A ==> T
	20235	1	0.333	C ==> T
	20863	1	0.231	G ==> C
	21030	1	0.143	C ==> T
	21137	1	0.167	T ==> C
	21290	1	1.000	C ==> A
	21495	1	0.182	T ==> G
	21755	1	0.273	C ==> G
	21926	1	1.000	A ==> C
	22056	1	0.600	T ==> A
	22490	1	1.000	C ==> T
	22722	1	0.250	G ==> C
	22763	1	1.000	C ==> G
	22961	1	1.000	T ==> C
	23024	1	1.000	C ==> A
	23298	1	0.167	C ==> A
	23503	1	1.000	G ==> A
	23628	1	0.429	G ==> C
	24640	1	1.000	G ==> A
	24774	1	1.000	G ==> C
	24937	1	0.667	C ==> G
	25071	1	1.000	C ==> A
	25331	1	1.000	C ==> G
	26288	1	0.400	G ==> T
node_112 --> MH539766 SADSCoV CHN GD 2017	238	1	0.100	C ==> T
	255	1	0.333	A ==> G
	301	1	0.500	G ==> T
	336	1	0.250	T ==> C
	437	1	0.200	T ==> C
	453	1	0.143	T ==> C
	501	1	0.500	C ==> T
	546	1	0.200	C ==> T
	558	1	0.182	T ==> C
	570	1	0.200	C ==> T
	600	1	0.143	C ==> T
	603	1	0.222	A ==> T
	606	1	0.167	G ==> A
	628	1	0.167	A ==> G
	633	1	0.100	T ==> C
	637	1	0.143	T ==> C
	642	1	0.143	C ==> T
	657	1	0.500	C ==> T
	717	1	0.500	C ==> T
	764	1	0.333	T ==> C
	765	1	0.429	A ==> G
	780	1	0.333	C ==> T
	783	1	0.111	T ==> C
	824	1	0.500	C ==> T
	834	1	0.125	C ==> T
	837	1	0.500	C ==> T
	910	1	0.250	A ==> G
	930	1	0.077	T ==> C
	933	1	0.250	T ==> C
	984	1	0.091	T ==> C
	987	1	0.429	A ==> G
	997	1	0.333	C ==> A
	1027	1	0.500	A ==> G
	1040	1	0.500	A ==> G
	1074	1	0.167	T ==> C
	1080	1	0.333	G ==> A
	1096	1	0.167	T ==> G
	1097	1	0.250	C ==> T
	1098	1	0.600	C ==> T
	1112	1	0.250	C ==> T
	1113	1	0.429	G ==> A
	1119	1	0.222	G ==> A
	1128	1	0.333	C ==> T
	1137	1	0.429	T ==> G
	1140	1	0.750	G ==> T
	1152	1	0.154	C ==> T
	1158	1	0.200	C ==> T

	1163	1	0.400	T ==> C
	1173	1	0.333	T ==> C
	1179	1	0.333	C ==> A
	1284	1	0.333	T ==> C
	1338	1	0.100	C ==> T
	1371	1	0.100	C ==> T
	1392	1	0.200	C ==> T
	1408	1	0.500	T ==> G
	1431	1	0.167	T ==> C
	1449	1	0.231	C ==> T
	4317	1	0.200	C ==> T
	5706	1	0.200	T ==> C
	11634	1	0.250	C ==> T
	13587	1	0.250	G ==> T
	14493	1	0.500	T ==> A
	20905	1	0.125	T ==> A
	20926	1	0.176	A ==> G
	21358	1	0.154	A ==> C
	21646	1	0.250	T ==> A
	24039	1	0.143	A ==> G
	24042	1	0.273	A ==> G
	24060	1	0.143	C ==> T
	24063	1	0.111	T ==> C
	24495	1	0.200	T ==> C
	24499	1	0.333	T ==> C
	24501	1	0.250	A ==> C
	24502	1	0.333	T ==> G
	27197	1	0.500	C ==> T
	27199	1	0.400	A ==> T
	27203	1	0.333	T ==> G
	27207	1	0.200	T ==> C
node_112 --> MK977618 SADSCoV SeACoV p10	179	1	0.667	G ==> T
	1305	1	0.200	C ==> T
	6801	1	0.333	A ==> G
	23113	1	1.000	A ==> C
	24701	1	1.000	T ==> G
	24904	1	0.200	C ==> T
	25986	1	0.667	G ==> T
node_114 --> MT199592 SADSCoV GDZJ02 2018	6216	1	0.400	A ==> C
	7788	1	1.000	T ==> G
	11136	1	1.000	T ==> G
	11159	1	1.000	T ==> G
	11958	1	0.143	C ==> T
	15324	1	0.250	C ==> A
	15338	1	1.000	C ==> G
	23052	1	0.300	A ==> C
node_115 --> MF769441 SADSCoV 82	10875	1	0.500	T ==> C
node_126 --> MF094682 SADSCoV FarmB	35	1	0.048	C ==> T
	2949	1	0.143	T ==> C
node_126 --> MF094683 SADSCoV FarmC	35	1	0.048	C ==> T
node_118 --> MF094684 SADSCoV FarmD	2230	1	0.500	G ==> T
node_118 --> MF769418 SADSCoV 207	2110	1	0.250	C ==> T
node_118 --> MK651076 SADSCoV GDLX 2019	35	1	0.048	T --> C
	3279	1	0.250	T ==> G
	21434	1	1.000	A ==> T
	23191	1	0.167	C ==> A
	25786	1	1.000	C ==> T
node_117 --> MT747186 SADSCoV CN GDDCD 2017	4449	1	0.500	A ==> C
	21137	1	0.167	C ==> T
	21578	1	1.000	G ==> C
	23044	1	1.000	G ==> T
node_117 --> MT747187 SADSCoV CN GDDE 2017	22543	1	0.333	A ==> G
	23026	1	1.000	A ==> C
node_126 --> MF167434 SADSCoV GDS04	1	1	0.333	G ==> C
	2949	1	0.143	T ==> C
	4567	1	0.200	G ==> A
node_119 --> MK994935 SADSCoV GDWT P18	677	1	1.000	C ==> T
	10752	1	0.600	A ==> C
	24904	1	0.200	C ==> T
node_126 --> MF769428 SADSCoV 53	35	1	0.048	C ==> T
	22041	1	0.188	G ==> C
	22236	1	0.143	C ==> T
node_126 --> MG605090 SADSCoV CN GDGL 2017	5011	1	0.333	T ==> C
	5081	1	1.000	A ==> G
	7252	1	0.500	T ==> C
	20872	1	0.231	G ==> T
	23280	1	0.600	T ==> A
	25002	1	0.667	T ==> C
	25068	1	1.000	T ==> C
node_126 --> MG605091 SADSCoV CN GDLS 2017	525	1	1.000	C ==> T
	764	1	0.333	T ==> C
node_123 --> MT199591 SADSCoV GDGL01 2016	3	1	1.000	C ==> A
	4	1	1.000	T ==> C
	7724	1	1.000	C ==> A
	22003	1	0.500	C ==> T
node_123 --> MT747188 SADSCoV CN GDST 2017	1848	1	0.200	C ==> T
	23047	1	1.000	A ==> G
node_124 --> OK078017 SADSCoV CH JX2 2018	13968	1	0.429	T ==> G
	21137	1	0.167	C ==> T
	21505	1	0.500	C ==> T
	23573	1	1.000	A ==> G
	24094	1	1.000	A ==> C
node_124 --> OK078018 SADSCoV CH JX1 2018	15411	1	0.222	C ==> T
	23203	1	1.000	T ==> C
	24683	1	1.000	T ==> C
node_126 --> MW727454 SADSCoV GDZQ 2018	1	1	0.333	G ==> C
	2949	1	0.143	T ==> C
	6619	1	1.000	G ==> A
	24904	1	0.200	C ==> T
node_127 --> ON911569 SADSCoV Guangxi 2021	62	1	1.000	T ==> A
	98	1	1.000	A ==> C
	253	1	1.000	T ==> A
	20550	1	0.429	T ==> A
	20815	1	0.400	C ==> A
	21062	1	1.000	C ==> G
	21113	1	0.250	A ==> G
	21114	1	0.300	T ==> A
	21115	1	0.667	A --> C
	21116	1	1.000	G ==> T

22250	1	0.333	T ==> C
22252	1	0.500	G ==> A
22253	1	1.000	A ==> C
22254	1	0.125	C ==> T
22255	1	1.000	C ==> T
22403	1	1.000	T ==> G
22418	1	0.500	T ==> C
22474	1	0.375	T ==> C
22556	1	0.667	A ==> G
22630	1	1.000	T ==> A
22827	1	0.200	A ==> T
22846	1	1.000	C ==> G
22964	1	1.000	T ==> G
23030	1	1.000	G ==> A
23042	1	0.500	A ==> G
23718	1	0.500	T ==> A
23725	1	0.500	T ==> G
24006	1	0.273	A ==> C
24117	1	0.250	G ==> A
24370	1	1.000	A ==> C
24458	1	1.000	T ==> C
24598	1	0.667	A ==> G
24918	1	0.111	C ==> T
24923	1	1.000	G ==> C
24924	1	0.400	C ==> A
24942	1	0.400	A ==> G
24943	1	0.333	G ==> A
25139	1	1.000	T ==> G
25158	1	1.000	T ==> A
25187	1	1.000	T ==> A
25244	1	1.000	C ==> T
25457	1	1.000	T ==> G
26692	1	0.500	T ==> C
26695	1	1.000	A ==> G
26743	1	0.333	T ==> A

Figure S9. List of autapomorphies found in SADS-CoV genomes based on a strict consensus of 672 most-parsimonious trees.

Number of autapomorphies (in grey) and exclusive autapomorphies (in red) for each of the 34 SADS-CoV genomes. The GenBank accession number of each genome is indicated in abscissa. The whole-genome alignment of 95 sequences and 27,250 nucleotides was executed in PAUP 4.0a (Swofford, 2021) to found the most-parsimonious (MP) trees by heuristic search. Then, the 672 MP trees of 43,156 steps were used to construct the strict consensus tree (data not shown) and the number of autapomorphies and exclusive autapomorphies (unique nucleotide substitutions characterised by consistency index = 1) found for each of the 34 SADS-CoV sequences was counted. The consensus tree and list of autapomorphies (position in the alignment, consistency index and substitution type) are available upon request.

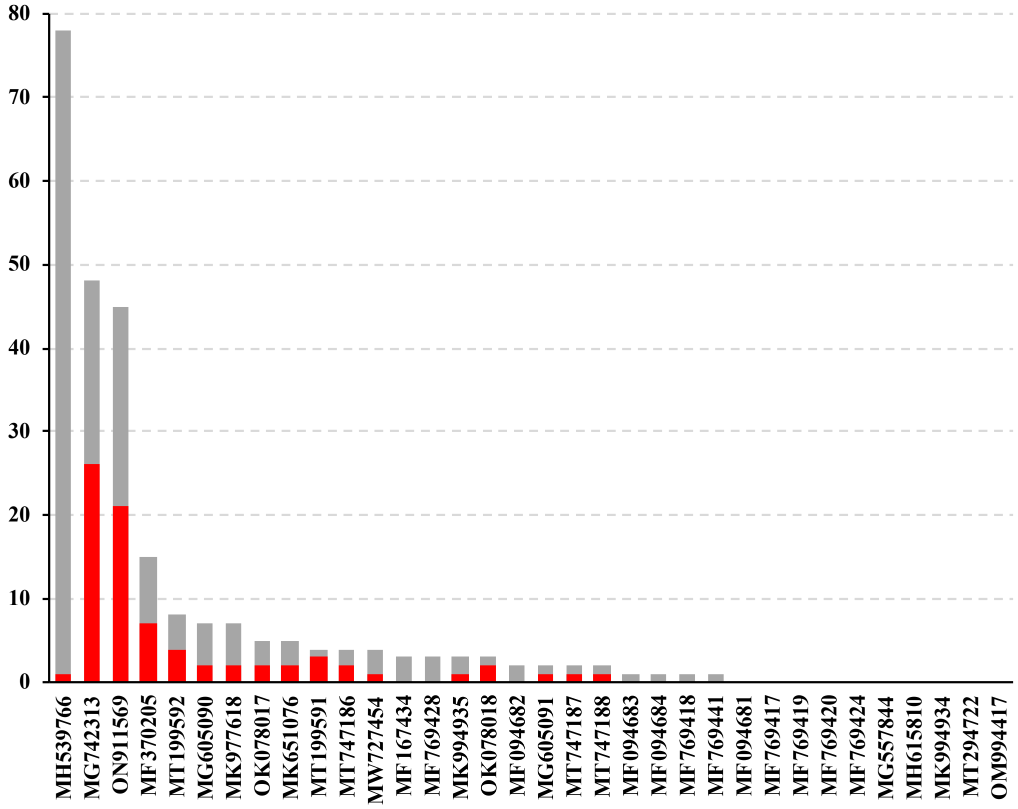

Figure S10. Maximum-likelihood tree of 87 *Rhinacovirus* genomes. The six genomes identified as problematic (see Results section 3.4 for more details) were excluded from the original analysis and the reduced dataset was analysed using IQtree (Nguyen et al., 2015) and 1,000 bootstrap replicates. Nodes consistent with the SuperTRI consensus in **figure S10** are indicated by thicker branches.

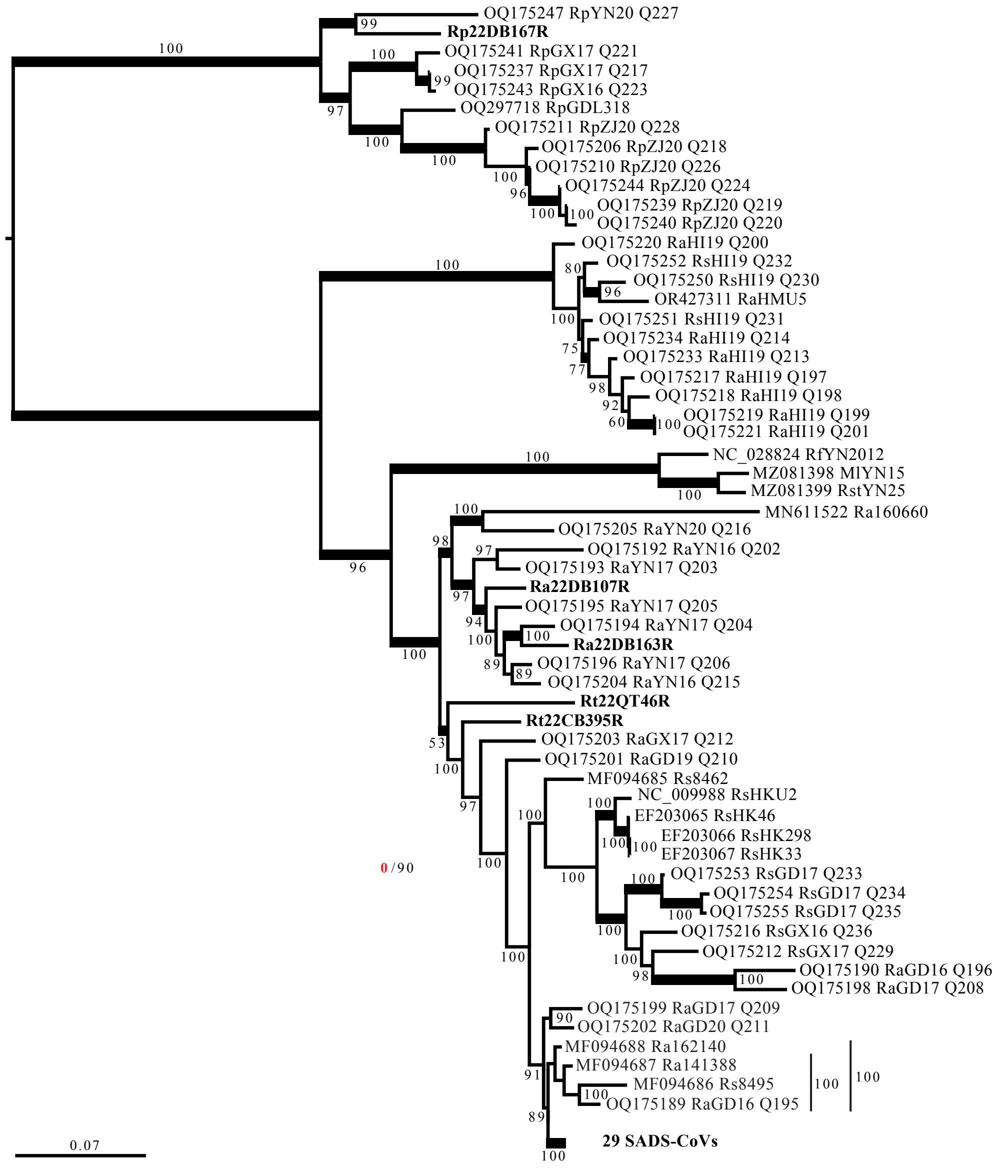

Figure S11. SuperTRI consensus tree built from five SWB analyses based on an alignment of 87 *Rhinacovirus* genomes. The alignment of 27,250 nucleotides was analysed using the SWB program (Hassanin et al., 2022) and five different window sizes (400, 500, 600, 1000 or 2000 nt). Then, the five SWB output files were transformed into five MRP files (with LFG and SuperTRI programs; Ropiquet et al., 2009; Hassanin et al., 2022) which were then executed in the PAUP 4.0 (Swofford, 2021) to construct five SuperTRI bootstrap 50%majority-rule consensus (SB) supertrees using weighted parsimony and 1,000 bootstrap replicates. The tree is a strict consensus of the five SB supertrees based on different window sizes.

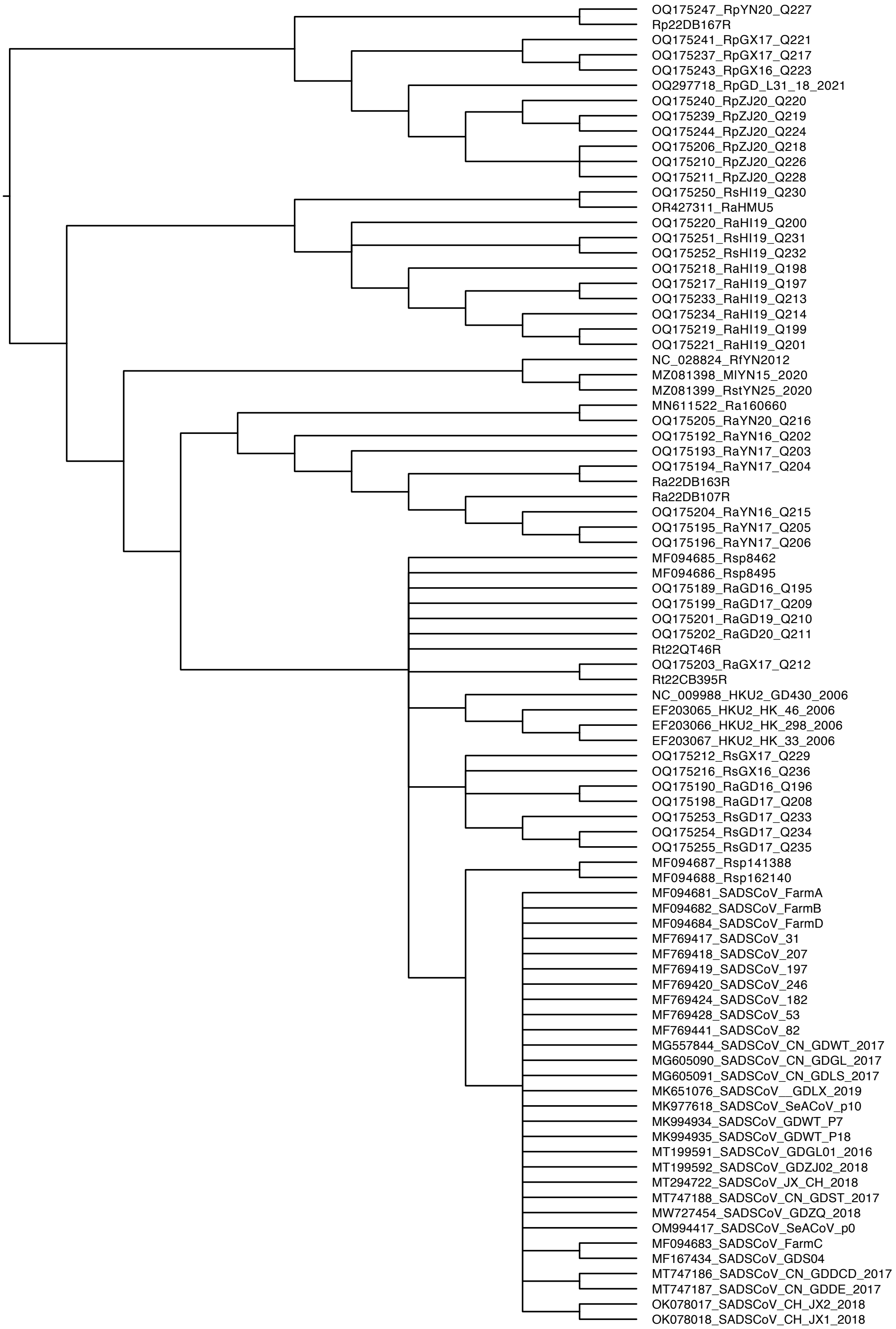

Supplement: Supplementary file 1 [file viruses-16-01114-s001.zip › Supplementary_figures.pdf]
