# Supplementary material for: Bat Rhinacoviruses Related to Swine Acute Diarrhoea Syndrome Coronavirus Evolve under Strong Host and Geographic Constraints in China and Vietnam
Source: Viruses. 2024 Jul 11;16(7):1114. doi: 10.3390/v16071114 (PMC11281452; doi:10.3390/v16071114)
Supplement: Supplementary file 1 [file viruses-16-01114-s001.zip › Table_S2_SWB_analyses.pdf]

Table S2. Five SWB analyses based on an alignment of 95 *Rhinacovirus* genomes (length: 27,250 nt)

	Window size (nt)	Step (nt)	WB sub-datasets	SWB bipartitions[1]	SuperTRI matrix[2]	BBC Bipartitions[3]	SADS-CoV bipartitions[4]
**1**	400	50	537	780,729	1,446,681	1,252	293
**2**	500	50	535	733,606	1,373,578	1,170	278
**3**	600	50	533	700,900	1,323,336	1,115	273
**4**	1000	50	525	618,380	1,186,335	986	246
**5**	2000	50	505	521,701	1,020,398	785	190

1: Number of bipartitions (with window bootstrap percentages [WBP] calculated for each WB subdatasets) obtained under the SWB program (Hassanin et al., 2022) [17,22];
2: Number of characters in the MRP matrix reconstructed using LFG (Hassanin et al., 2022) [17,22] and SuperTRI (Ropiquet et al., 2009) [23] programs;
3: Number of SWB bipartitions including at least one WBP ≥ 50% selected under the BBC program using the SWB file as input (Hassanin et al., 2022) [17,22];
4: Number of BBC bipartitions including the 34 SADS-CoV viruses.
