# Supplementary material for: Bat Rhinacoviruses Related to Swine Acute Diarrhoea Syndrome Coronavirus Evolve under Strong Host and Geographic Constraints in China and Vietnam
Source: Viruses. 2024 Jul 11;16(7):1114. doi: 10.3390/v16071114 (PMC11281452; doi:10.3390/v16071114)
Supplement: Supplementary file 1 [file viruses-16-01114-s001.zip › Table_S3_BLAST.pdf]

Table S3. Three top hits found by BLAST search of Vietnamese *Rhinacovirus* genomes in NCBI (https://blast.ncbi.nlm.nih.gov/).

Virus	Nucleotide Identity*	Query Cover (QC)	Virus name	GenBank accession
**Ra22DB107R**	96.86%	100%	RaYN17-Q205	OQ175195
	96.81%	100%	RaYN17-Q206	OQ175196
	96.54%	100%	RaYN16-Q215	OQ175204
**Ra22DB163R**	98.35%	98%	RaYN16-Q215	OQ175204
	96.68%	100%	RaYN17-Q204	OQ175194
	96.42%	100%	RaYN17-Q206	OQ175196
**Rp22DB167R**	92.84%	100%	RpYN20-Q227	OQ175247
	92.39%	100%	RpGX17-Q217	OQ175237
	92.38%	100%	RpGX16-Q223	OQ175243
**Rt22CB395R**	95.26%	100%	RaGX17-Q212	OQ175203
	94.99%	100%	RaYN17-Q205	OQ175195
	94.91%	100%	Rs8462	MF094685
**Rt22QT46R**	94.51%	98%	RaYN16-Q215	OQ175204
	94.48%	98%	RaGX17-Q212	OQ175203
	94.43%	98%	RaGD19-Q210	OQ175201

*: with QC ≥ 98%
